# The Sarcomeric M-Region: A Molecular Command Center for Diverse Cellular Processes

**DOI:** 10.1155/2015/714197

**Published:** 2015-04-15

**Authors:** Li-Yen R. Hu, Maegen A. Ackermann, Aikaterini Kontrogianni-Konstantopoulos

**Affiliations:** Department of Biochemistry and Molecular Biology, School of Medicine, University of Maryland, 108 N Greene Street, Baltimore, MD 21201, USA

## Abstract

The sarcomeric M-region anchors thick filaments and withstands the mechanical stress of contractions by deformation, thus enabling distribution of physiological forces along the length of thick filaments. While the role of the M-region in supporting myofibrillar structure and contractility is well established, its role in mediating additional cellular processes has only recently started to emerge. As such, M-region is the hub of key protein players contributing to cytoskeletal remodeling, signal transduction, mechanosensing, metabolism, and proteasomal degradation. Mutations in genes encoding M-region related proteins lead to development of severe and lethal cardiac and skeletal myopathies affecting mankind. Herein, we describe the main cellular processes taking place at the M-region, other than thick filament assembly, and discuss human myopathies associated with mutant or truncated M-region proteins.

## 1. Introduction

**Figure 1:**
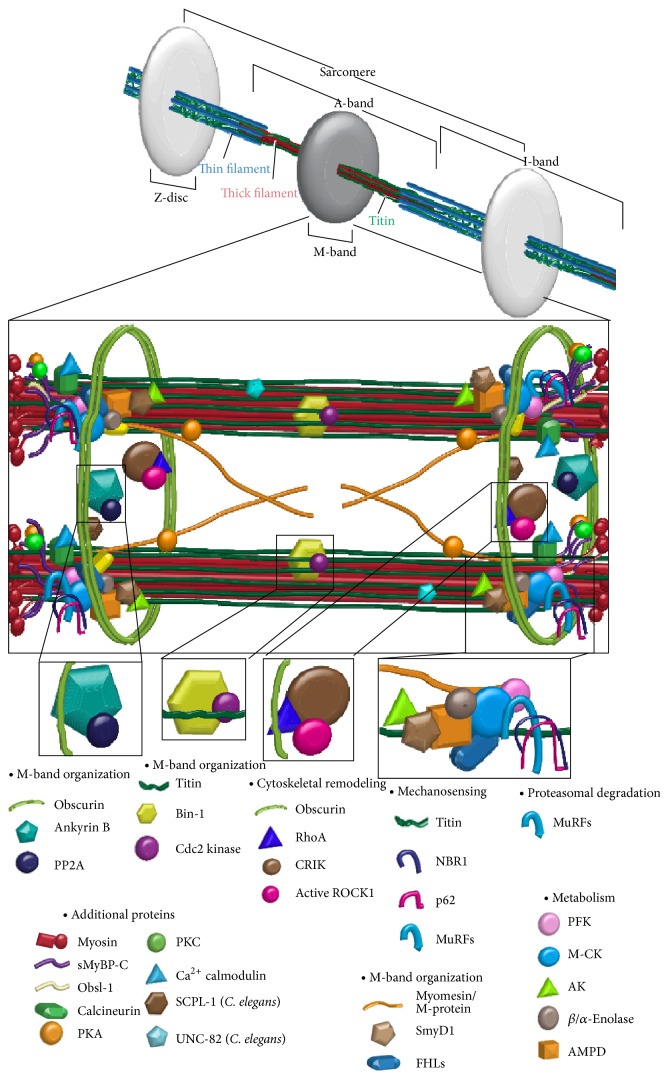
Sarcomeric M-region road map and cellular processes. Schematic representation of the sarcomeric M-region depicting key proteins and highlighting cellular processes.

The M-band is a dense protein-packed structure at the center of the A-band of cardiac and skeletal muscle cells (Figure 1 and Table 1). Under the electron microscope, M-band appears as a series of dark transverse lines spanning ~500–750 Å, depending on fiber type and species [1]. Named for the German word “mittelscheibe,” which means “central disc,” the M-band lies at the center of the bare zone, which is devoid of myosin heads and cross-bridges [2] but encompasses overlapping arrays of antiparallel myosin rods [3]. Adjacent myosin rods are connected via M-bridges, forming a regular hexagonal lattice [4]. The M-bridges are necessary to maintain thick filament alignment and aid in the controlled distribution of mechanical stress across the sarcomere during active contraction [5].

In addition to the rod region of myosin, the M-band is “home” to several other proteins (Figure 1 and Table 1). As such, the COOH-termini of titin molecules from half sarcomeres converge in an antiparallel fashion at the M-band [6]. Composed of immunoglobulin (Ig) domains and unique sequences, the COOH-terminus of titin is located downstream of its kinase domain, which is found at the junction of A- and M-bands.

The M-band also contains myomesin, M-protein, and myomesin-3, which share similar domain architectures and are primarily composed of Ig and fibronectin type III (FnIII) domains, but contain distinct NH_2_-terminal heads [7, 8]. These proteins are the principal components of M-bridges forming the backbone of the M-band filamentous system, which cross-links neighboring thick filaments [6, 8].

Also localizing at the level of the M-region are additional sarcomeric and membrane associated proteins, including obscurins, select variants of Myosin Binding Protein-C Slow, ankyrins, and spectrins [9–12]. These contribute to the assembly and stabilization of the M-region and its linkage with the sarcomeric cytoskeleton, the sarcoplasmic reticulum (SR), and the sarcolemma [13].

Within the last decade, our knowledge of the sarcomeric M-region has steadily expanded. To date, there are several excellent reviews on protein complexes mediating the assembly and organization of the entire M-region encompassing the M-band core as well as its periphery, and its role in thick filament assembly and integration into A-bands [5, 14]. Herein, we focus on key protein mediators of additional cellular processes occurring at the M-region. In addition, we highlight skeletal and cardiac myopathies that are linked to mutations in genes encoding M-region related proteins.

## 2. Cellular Processes at the M-Region

M-region is the hub for multiple cellular processes including signal transduction, metabolism, mechanosensing, and proteasomal degradation. Such processes support cellular homeostasis, myofibrillar organization, and contractile activity by maintaining sarcomeric integrity, meeting the energy demand during active contraction, and enabling adaptation to different biochemical and biomechanical stimuli. Below we will address these important processes occurring at the M-region and discuss key proteins.

### 2.1. Signal Transduction via Posttranslational Modifications

Two main types of posttranslational modifications, phosphorylation and sumoylation, have been described at the M-region. These mediate proper protein localization, regulate protein-protein interactions, and relay signals in response to biochemical or biomechanical stimuli.

#### 2.1.1. Phosphorylation

Several M-region proteins possess active kinase domains and/or are regulated by phosphorylation. Below we discuss such proteins.

*Titin (~3-4 MDa).* The giant protein titin extends longitudinally across a half-sarcomere, with its NH_2_-terminus anchored to the Z-disc, and its COOH-terminus localized at the center of the M-band [15]. The M-band portion of titin (~200 kDa) is composed of ten Ig CII type domains, which are interspersed with unique nonmodular segments, termed M-insertions [13, 16, 17]. The insertion between Ig CII-5 and Ig CII-6 contains tandem lysine-serine-proline (KSP) repeats, which are heavily phosphorylated by Cdc2 kinase in developing, but not in differentiated muscle cells [16]. The phosphorylated KSP repeats interact* in vitro *with the SH3 domain of the tumor suppressor bridging integrator protein 1 (Bin1) [18]. Bin1 is a negative regulator of c-Myc activation that is preferentially expressed in differentiating, but not mature, myotubes [18]. Transgenic mice overexpressing the SH3 domain of Bin1 exhibit dramatic disarray in myofiber size and structure [18]. Conversely, mouse C2C12 skeletal myoblasts fail to differentiate following downregulation of Bin1 [19, 20], and Bin1 homozygous knock-out mice develop hypertrophic cardiomyopathy leading to perinatal lethality, although skeletal muscles do not exhibit any apparent abnormalities at this stage [21]. It is therefore possible that disruption of the interaction between the phosphorylated KSP repeats of titin and Bin1 at the M-band may result in deregulated myofibrillar assembly, loss of differentiation, and aberrant fiber size.

In addition to being phosphorylated within its M-band portion, titin may regulate different sarcomeric processes via its kinase domain, residing at the periphery of the M-band [6, 13, 22, 23]; one of these processes is mechanosensing, which will be discussed in a later section of this review.

*Obscurin (720–890 kDa). *Incorporated early during development into the periphery of the sarcomeric M-band, obscurin is an important player of thick filament assembly and stabilization [24]. Downregulation of obscurin using siRNA technology leads to selective disorganization of M- and A-bands in developing skeletal and cardiac myocytes, indicating its scaffolding role [24–26]. Importantly, recent studies have demonstrated that the two Ser/Thr kinase domains, which are present at the COOH-terminus of giant obscurin-B are catalytically active and can directly bind major components of the M-band, including titin and four and a half LIM domains 2 (FHL2) protein [27, 28]. Examining if these proteins are catalytic substrates of the obscurin kinase domains at the M-band will be the next challenge.

Obscurin is also involved in dephosphorylation events at the M-band. Ankyrin-B (Ank-B), fulfilling its role as an anchoring protein, binds directly to both obscurin and the regulatory subunit, B56*α*, of protein phosphatase 2A (PP2A) at the M-band [29, 30]. Overexpression of the obscurin-binding region of Ank-B in isolated adult cardiomyocytes results in displacement of B56*α* from the M-band [29]. Moreover, UNC-89, the obscurin homologue in* C. elegans*, directly binds to small C-terminal domain (CTD) phosphatase-like 1 (SCPL-1) at the M-band [28, 31]. However, the cellular processes that PP2A and SCPL-1 mediate at the M-band are still unknown.

*Myomesin (178–188 kDa).* Spanning the entire M-region by residing in the interior yet extending to the periphery of the M-band, myomesin serves as a cross-linker between myosin, titin, and obscurin [32, 33]. It consists of tandem Ig and FnIII domains, along with a nonmodular NH_2_-terminus [32–35]. While the NH_2_-terminus of myomesin interacts with sarcomeric myosin [36], the region encompassing Ig domains 4 and 5 (My4-My5) directly interacts with titin Ig4 within its M-band portion (MIg4) [32, 34]. Phosphorylation of the linker region between myomesin domains My4 and My5 by protein kinase A (PKA) abolishes its binding to titin MIg4 [32]. Importantly, the linker region also interacts with two other M-band proteins, obscurin, and obscurin-like 1, but these interactions are not modulated by phosphorylation [33]. Thus, a ternary complex of regulated and potentially constitutive interactions between myomesin and titin, and myomesin and obscurin, or obsl-1 occurs at the M-band [33].

*M-Protein (~165 kDa). *Similar to myomesin, M-protein also consists of tandem Ig and FnIII domains, which are preceded by a unique NH_2_-terminus [6, 37, 38]. Unlike myomesin though, which is expressed ubiquitously in all striated muscles, M-protein is only expressed in cardiac and fast-twitch skeletal muscles [39–41]. Phosphorylation at S76, present in the nonmodular NH_2_-terminus of M-protein, by PKA abolishes its binding to myosin [42].

Thus, the PKA-mediated phosphorylation of both myomesin and M-protein may modulate thick filament integration, organization, and stability at the M-band during myofibrillogenesis or myofilament turnover.

Myomesin-3 (~162 kDa), also composed of a nonmodular NH_2_-terminus and tandem Ig and FnIII domains, is present at the M-band, too [8]. Unlike myomesin, which is ubiquitously present in all striated muscles and M-protein that is preferentially expressed in cardiac and fast-twitch skeletal muscles, myomesin-3 is selectively expressed in slow-twitch skeletal muscles [8, 43]. Whether myomesin-3 is subjected to posttranslational modifications, however, remains to be investigated. Along the same lines, select variants of Myosin binding protein-C slow (MyBP-C slow), FHL1, and FHL2 and obscurin-like 1 also localize at the M-region [10, 33, 44–48]. Out of these proteins, at least MyBP-C slow is modulated via phosphorylation mediated by PKA and PKC [49]. Moreover, the Ca^2+^/calmodulin-dependent serine/threonine phosphatase, calcineurin (also known as protein phosphatase 2B, PP2B), is also present at the M-band and may mediate dephosphorylation events of M-band proteins [47]. At this time though, the physiological significance of the phosphorylation and dephosphorylation events involving these proteins remains speculative.

In addition to the phosphorylation events discussed above, which have been mainly studied in mammalian striated muscles, additional protein players involved in posttranslational modification events within the M-region have been reported in* C. elegans*. Unlike the cross-striated muscles of mammals, myofilaments of body muscles in* C. elegans* are arranged in oblique striations. Therefore, both the protein composition and the functional role(s) of individual proteins at the M-region of body muscles of* C. elegans* may differ from those of mammalian striated muscles. Below, we provide such an example.

*UNC-82 (33–203 kDa)*. Recently identified as a Ser/Thr kinase that localizes at or near the M-band in* C. elegans*, UNC-82 is closely related to mammalian proteins AMPK-related protein kinase 5 (ARK5) and sucrose nonfermenting AMPK-related kinase (SNARK) (also known as NUAK1 and NUAK2, resp.), which are members of the AMP-activated protein kinase (AMPK) family of Ser/Thr kinases [50]. While the enzymatic activity and substrates of UNC-82 remain to be determined, mutant* C. elegans* embryos containing either a missense mutation presumably abolishing its kinase activity or a nonsense mutation resulting in truncated UNC-82 exhibit disorganized A- and M-bands [50]. Interestingly, its closely related mammalian counterparts, ARK5 and SNARK, have been implicated in glucose metabolism in skeletal muscle [51, 52]; however the localization of ARK5 and SNARK has yet to be examined.

#### 2.1.2. SUMOylation

In addition to phosphorylation, proteins at the M-region undergo sumoylation. While less studied compared to phosphorylation, sumoylation has been implicated as a possible mechanism for targeting proteins to the M-region.

*Myomesin (178–188 kDa)*. In addition to being regulated via phosphorylation, myomesin is also subjected to sumoylation, possibly at K228, in adult rat cardiomyocytes [53]. Moreover, in neonatal rat ventricular myocytes (NRVM), where myomesin is localized to the nucleus, overexpression of the SUMO peptide mediates its translocation to the cytoplasm and promotes sarcomeric organization [54]. Sumoylation of myomesin is mediated by myofibrillogenesis regulator-1 (MR-1), which is highly expressed in both skeletal and cardiac muscles [54, 55]. While overexpression of MR-1 in mice enhances cardiac hypertrophy stimulated by angiotensin II, overexpression of MR-1 in NRVM induces sarcomeric organization and translocation of myomesin from the nucleus to the cytoplasm, similar to SUMO overexpression [54, 56]. Consistent with this, downregulation of MR-1 abolishes SUMO-induced translocation of myomesin to the cytoplasm and sarcomeric organization [54].

*SET and MYND Domain Containing-1 (SmyD1) (~54–56 kDa). *SmyD1 is another M-band protein whose localization is modulated by sumoylation [57, 58]. Also known as Bop, SmyD1 is a histone methyltransferase that is abundantly expressed in striated muscles with its methyl transferase activity attributed to its Su(var)3-9, enhancer-of-zeste, and trithorax (SET) domain [59–63]. In mouse, the* smyd1 *locus encodes two alternatively spliced isoforms, SmyD1_tv1, and SmyD1_tv2 (also known as skm-Bop1 and skm-Bop2, resp.) [60, 61, 63]. SmyD1_tv1 and SmyD1_tv2 localize at the M-band and the nucleus in both cardiac and skeletal muscles [57, 60]. Homozygous deletion of* smyd1* in mice results in embryonic lethality at E10.5 due to disrupted ventricular formation, which is accompanied by loss of right ventricles [61]. Recently, skeletal muscle- and heart-specific *α* nascent polypeptide-associated complex, skNAC, was shown to mediate the sumoylation of SmyD1 proteins and thus regulate their nuclear export and translocation to the M-band during sarcomerogenesis [58, 64]. Thus, sumoylation mediates SmyD1 targeting to the M-band, where it potentially regulates the activities of major M-band proteins, such as myosin and muscle-type creatine kinase, via its methyl transferase activity.

Proteins at the M-region undergo additional posttranslational modifications, such as acetylation, methylation, and neddylation. For instance, small ankyrin 1.5 (sAnk1.5) is subjected to acetylation and neddylation [65, 66], while muscle-type creatine kinase and myosin are subjected to acetylation and methylation [66, 67]. However, the functional significance and the identity of the relevant enzymes carrying out these posttranslational modifications at the M-band remain to be further investigated.

### 2.2. Cytoskeletal Remodeling via Small GTPases

Small GTPases play important roles in diverse cellular and developmental processes, including cytoskeletal remodeling, actomyosin contractility, vesicle transport, growth, and proliferation [68–72]. Small GTPases are regulated via repeating cycles of GTP binding and hydrolysis. Three accessory proteins contribute to their regulation: (i) GTPase activating proteins (GAP), which hydrolyze GTP to GDP, inactivating small GTPases, (ii) guanine nucleotide exchange factors (GEF), which mediate the exchange of GDP to GTP activating small GTPases, and (iii) guanosine nucleotide dissociation inhibitors (GDI), which prevent the exchange of GDP for GTP by sequestering GTPases and preventing them from binding to downstream effectors [73, 74]. Ras homolog gene family, member A protein, RhoA, localizes at the M-band in adult skeletal and cardiac muscles [66, 75]. While RhoA mediates several cellular processes, the discussion below will mainly focus on its roles in cytoskeletal remodeling at the M-band.

#### 2.2.1. RhoA Signaling in Skeletal Muscle

Inactive RhoA preferentially localizes to the M-band of adult skeletal myofibers, whereas active RhoA exhibits a dual distribution, at both M-bands and Z-disks [75]. RhoA activity is significantly increased following overexpression of the obscurin RhoGEF motif in adult rat tibialis anterior (TA) muscle and after injury induced by large-strain lengthening contractions [75]. Consistent with this, the RhoGEF/Pleckstrin homology cassette of Unc-89, the* C. elegans* obscurin homologue, activates Rho-1, the RhoA* C. elegans* homologue [76]. In mammalian skeletal muscle, active RhoA leads to loss of citron rho-interacting kinase (CRIK), which is involved in the regulation of cytokinesis in proliferating cells, from M- and A-bands [75, 77, 78]. Moreover, active RhoA leads to translocation of Rho-associated protein kinase 1 (ROCK1) from the Z-disk to the I-band, the Z/I junction, and the M-band [75]. Although the functional ramifications of the concurrent loss of CRIK from the M-band and the translocation of ROCK1 to the M-band are still elusive, it is likely that they mediate the activation of stretch-response genes leading to cytoskeletal remodeling or the development of hypertrophy following injury. Consistent with this, activation of the RhoA/ROCK pathway in dystrophin/utrophin double knockout mice has been implicated with heterotopic ossification, while inhibition of the RhoA/ROCK pathway has been associated with improved myogenic potential [79, 80].

RhoA localizes at both M-bands and Z-disks in cardiac muscle, too, although its distribution has not been correlated with its state of activation [66]. Extensive studies have focused on the diverse processes that RhoA mediates in the developing and adult myocardium, emphasizing its roles in the regulation of actin filament assembly and stress fiber formation, sarcomeric organization, induction of a hypertrophic response, tolerance to ischemia/reperfusion, survival, and apoptosis [81–88]. Given that it is currently unknown whether RhoA contributes to these processes through its interactions at the Z-disk or the M-band, we will refrain from presenting such studies in detail.

### 2.3. Mechanosensing

In addition to biochemical stimuli, muscle cells respond to biomechanical stimuli by modulating protein expression through activation of signaling pathways [89, 90]. Consistent with this, both the Z-disk [91, 92] and the M-band [92] contain mechanosensors that may transform biomechanical stimuli to biochemical signals. At the M-band, the kinase domain of titin is a major player mediating cell responses to mechanical stress.

#### 2.3.1. Titin Kinase

Use of atomic force microscopy (AFM) has demonstrated that the kinase domain of titin is activated upon exertion of mechanical force, leading to unfolding of its regulatory autoinhibitory tail and phosphorylation of Y170, allowing ATP binding to the catalytic aspartate [93]. It is therefore likely that activation of the titin kinase via mechanical force may result in regulation of its proximal substrates via phosphorylation. This is exemplified in the case of the direct interaction between the titin kinase domain and the muscle-specific ring finger (MuRF) complex, consisting of NBR1 (neighbor of BRCA1 gene 1 protein)/p62/MURF-2, which has been suggested to regulate protein turnover in response to mechanical force [23]. Both NBR1 and p62 are substrates of titin kinase [23] and are required in autophagosome-mediated protein degradation by serving as receptors for ubiquitinated proteins [94–96]. Since NBR1 can only bind to the semiopened conformation of the titin kinase that is likely induced by exertion of mechanical force during stretching, it is possible that MuRF-2 is anchored to the M-band via the interaction of NBR1 with the kinase domain of titin [23]. Consistent with this, denervation of skeletal myofibers or mechanical arrest of neonatal cardiomyocytes leads to dissociation of the MuRF-2/NBR1/p62 complex, with MuRF-2 translocating to the nucleus in both skeletal and cardiac cells where it suppresses serum response factor- (SRF-) mediated expression of hypertrophic genes, and p62 accumulating at intercalated disks in cardiac cells [23]. It is noteworthy to mention, however, that a recent study indicated that the kinase domain of titin is not enzymatically active but serves as a scaffold for other proteins that localize to the M-region [97]. Additional studies are warranted to resolve these opposing results.

### 2.4. Metabolism

Sarcomeric M-band is strategically located in close proximity to where significant amounts of energy are consumed during repeating cycles of actomyosin contractility [98–100]. Maintaining the ATP and ADP levels at optimal concentrations in the sarcoplasm is essential in sustaining muscle activity, as depletion of ATP or accumulation of ADP would attenuate contractility [101]. Below we discuss several proteins localized at the M-band, which play key roles in metabolism by maintaining the ATP/ADP ratio at optimal levels within the sarcoplasm.

#### 2.4.1. Muscle-Type Creatine Kinase (M-CK)

Creatine kinase (CK) catalyzes the phosphate transfer from phosphocreatine to ADP, generating ATP and creatine (Phosphocreatine^2−^ + MgADP^−^ + H^+^↔ MgATP^2−^ + creatine) [102]. In the mammalian genome, there are four gene loci encoding the different CK isoenzymes: muscle-type CK (M-CK), brain-type CK (B-CK), ubiquitous mitochondrial CK (uMtCK), and sarcomeric mitochondrial CK (sMtCK) [103]. While both M-CK and sMtCK are readily expressed in striated muscles, only M-CK is present within the sarcomere [103–105]. M-CK functions as a dimer and localizes at the M-band by interacting with myomesin, M-protein, and FHL-2 [46, 106]. Homozygous null M-CK mice exhibit decreased voluntary running capability, which is accompanied by a significant reduction in force production during initial contractions due to inadequate supply of local ATP [105, 107–109]. Interestingly, malignant transformation of skeletal muscle to sarcoma results in reduction of M-CK levels, indicating that M-CK supports the specific metabolic needs of skeletal myofibers, which gradually lose their differentiation status and contractile properties during sarcoma development [110].

#### 2.4.2. Adenylate Kinase (AK)

Similar to M-CK, AK is also present at the M-band [46]. AK catalyzes the transfer of a phosphate from ADP to another ADP to generate AMP and ATP and* vice versa* (MgADP^−^ + ADP^2−^↔ MgATP^2−^ + AMP^−^), thus maintaining myofibrillar ATP and ADP concentrations at optimal levels [111, 112]. There are nine AK isoenzymes in mammals, referred to as AK1-AK9 [113]. While the majority of AKs localize to mitochondria, AK1 and AK7 are primarily present in the sarcoplasm [113]. At rest, skeletal muscles of homozygous AK1 knockout mice contain increased levels of AMP, without any other pathological phenotype [112]. However, upon induction of high frequency (90–120* tetani*/min) contractions, gastrocnemius, plantaris, and soleus AK1 null muscles exhibit increased levels of ADP, accompanied by slower relaxation rate, although the magnitude of the generated force is unaltered [112, 114, 115]. The slower relaxation rate is likely due to reduced Gibbs-free energy in response of the increased ADP : ATP ratio, resulting in compromised sarco/endoplasmic reticulum Ca^2+^-ATPase (SERCA) activity and decreased Ca^2+^ uptake by the SR [112]. Moreover, exposure of AK1 deficient mice to ischemia/reperfusion results in accelerated loss of cardiac contractility and reduced ATP and ADP levels during reperfusion, underscoring the importance of AK1 in supporting myocardial function by regulating energy metabolism [116]. The presence of AK in the sarcomere is therefore essential for meeting the high-energy demands of muscle cells and protecting them from insults.

#### 2.4.3. Adenosine Monophosphate Deaminase (AMPD)

Three genes encoding AMPD proteins have been identified:* AMPD1*,* AMPD2*, and* AMPD3 *(reviewed in [117]).* AMPD1* encodes an AMPD form that is preferentially expressed in striated muscles, M-AMPD [118, 119]. M-AMPD at the M-band is coupled with M-CK and AK to modulate the ATP, ADP, and AMP levels [101, 120]. AMPD catalyzes the removal of an amine group from AMP, giving rise to ammonia and IMP (AMP^−^ → IMP^−^ + NH_3_) [101]. In conjunction with AK, AMPD maintains constant intracellular ADP levels, therefore preventing AMP accumulation and favoring the formation of ATP by AK [101].

#### 2.4.4. Phosphofructokinase (PFK)

The fourth metabolic enzyme residing at the M-band, PFK, mediates the transfer of a phosphate group from ATP to fructose-6-phosphate to yield fructose-1, 6-bisphosphate (Fructose-6-P^−^ + MgATP^2−^ → Fructose-1,6-P_2_^2−^ + MgADP^−^), and is a rate-limiting enzyme of the glycolytic pathway [121, 122]. There are three PFK isoenzymes in mammals, PFK-M (muscle), PFK-L (liver), and PFK-P (platelet). Skeletal muscles express exclusively PFK-M, whereas cardiac muscle expresses all three isoenzymes, with PFK-M being the predominant one [123]. Fully activated PFK exists in tetrameric or a more complex oligomeric form, while dimeric PFK confers minimal activity [124]. PFK's activity is modulated by allosteric regulators (e.g., adenosine phosphates and fructose-2, 6-bisphosphate), interacting partners (e.g., F-actin and calmodulin), or posttranslational modifications (e.g., acylation and phosphorylation) [122, 124–126]. Thus, AMP and ADP stabilize PFK in a tetrameric conformation, whereas ATP and citrate stabilize PFK in a dimeric conformation [121, 124, 127–132]. Enhanced binding to F-actin in response to insulin stimulation also stabilizes the tetrameric form of PFK and maintains it in an active conformation [133–135]. Alternatively, PFK may be regulated by a complex mechanism in response to calcium fluctuation in the sarcoplasm [136]. Given the presence of two calmodulin binding sites in PFK, it has been proposed that PFK activity is strongly inhibited when both sites are occupied [124, 137]. However, occupation of only one calmodulin site may abrogate the inhibitory effect mediated by ATP- and citrate-binding via induction of a dimeric conformation that is fully active [124, 138]. Intriguingly, phosphorylation mediated by calmodulin-dependent kinase (CaMK) results in increased sensitivity to ATP inhibition [129]. Since calmodulin binding is modulated by Ca^2+^, the activity of PFK may be modulated by the Ca^2+^ levels in the sarcoplasm, especially during contractions. PFK-M homozygous null mice (PFKM^−/−^) exhibit high mortality at weaning (60%), reduced life-span (~3–6 months), and decreased ATP concentration in skeletal muscles, accompanied by increased glycogen content and exercise intolerance [139]. Notably, the small number of PFKM null animals that survive beyond 6 months of age develops cardiac hypertrophy by year one [139]. The reduced viability of the PFKM deficient mice is consistent with PFK being the rate-limiting enzyme in the glycolytic pathway and underscores its key role in energy production.

#### 2.4.5. Enolase

In addition to PFK, enolase is another glycolytic enzyme that resides at the M-band [140, 141]. Three gene loci encode the three known enolase isozymes, which are expressed in different tissues: nonneuronal enolase, *α (NNE)*, muscle-specific enolase, *β (MSE), *and neuronal-specific enolase, *γ (NSE)* [142, 143]. Both *α*- and *β*-isozymes are expressed in cardiac and skeletal muscles, localize at the M-band, and may form homo- or heterodimers [140, 141, 144, 145]. Dimeric enolase converts the glycolytic intermediate 2-phospho-D-glycerate to phosphoenolpyruvate (2-phospho-D-glycerate^2−^↔ phosphoenolpyruvate^2−^ + H_2_O) [146]. While *ββ*-enolase homodimers are predominantly expressed in skeletal muscle, especially in Type II muscle fibers [141, 147, 148], *αα*-, *αβ*-, and *ββ*-dimers are present in cardiac muscle [145, 148, 149]. The relative expression of *α* and *β* isozymes in the heart is important in fine-tuning metabolic activity. Consistent with this, in a rat hypertrophy model induced by aortic stenosis, where the rate of glycolysis is increased [150], the ratio of the *α*- to *β*-isozymes is increased in the heart, due to reduced expression of *β*-enolase, although the levels of *α*-enolase are unaltered [149]. Conversely, in the spontaneous hypertensive (SHR) rat model, the hypertrophied heart expresses increased levels of the *α*-isozyme, which is also hyperphosphorylated [149, 151, 152]. While the *α*- and *β*-enolases exhibit comparable enzymatic kinetics, the hyperphosphorylated *α*-isozyme performs slower catalysis [152, 153]. Overexpression of *α*-enolase in response to ischemia/reperfusion in a rat model confers improved contractility of affected cardiomyocytes [154]. Consistent with its protective role in the heart, *α*-enolase is significantly upregulated in mouse skeletal muscle, which predominately expresses *β*-enolase, in response to muscle injury induced by cardiotoxin; interestingly, the expression of *β*-enolase is drastically decreased after the first day but recovers a week later [155]. Similarly, in rat skeletal muscles subjected to denervation, the levels of the *αα* dimer are modestly increased, while the levels of the *ββ*-dimer are decreased [148]. It is noteworthy to mention that since *α*-enolase may also serve as a heat-shock protein or a plasminogen receptor involved in cardiac remodeling and muscle regeneration, it is possible that the protective role of *α*-enolase may not only be related to its glycolytic activity [152, 156].

Regulation of energy metabolism may also be mediated through proteasomal degradation. Muscle-specific RING finger proteins (MuRFs) present at the M-band ubiquitinate metabolic enzymes, which are subsequently targeted for proteasomal degradation [157–159]. Along these lines, oxidized muscle-type creatine kinase is rapidly ubiquitinated by MuRF-1 and subsequently targeted to the proteasome [159, 160]. In addition, MuRF-1 also interacts with adenylate kinase [157], although the effects of this interaction are still elusive. The importance of MuRFs at the M-band is further discussed in the following section.

### 2.5. Proteasomal Degradation

*MuRFs*. The MuRF family consists of three members, MuRF-1, MuRF-2, and MuRF-3, which are E3 ubiquitin ligases, and preferentially expressed in striated muscles [161–163]. They contain a RING finger domain at their NH_2_-terminus and transfer ubiquitin-chains to the proteins destined for proteasomal degradation [158, 162]. The poly-ubiquitinated proteins are recognized by the proteasome, subjected to deubiquitination, unfolding, and hydrolysis in its proteolysis core [164]. While MuRF-3 is ubiquitously expressed in skeletal and cardiac muscles, MuRF-1 is preferentially expressed in cardiac and fast-twitch skeletal muscles, and MuRF-2 is predominantly expressed in slow-twitch skeletal muscles, with minimal expression in cardiac and fast-twitch skeletal muscles [165, 166]. All three MuRFs localize to the M-band and the nucleus [167]. Additionally, MuRF-1 and MuRF-3 are also present at the Z-disc [167].

#### 2.5.1. MuRF-1

MuRF-1 interacts with the Ig168/Ig169 domains within the A-band portion of titin, while the presence of the adjacent titin kinase domain enhances this interaction [97, 163]. Overexpression of MuRF-1 disrupts the organization of M- and A-bands, but not of Z-disks and I-bands, in chick cardiomyocytes [168], indicating that increased protein turnover rate at M- and A-bands results in dissolution of these structures. Interestingly, MuRF-1 deficient mice exhibit no major alterations in the levels of ubiquitination within the myocardium, suggesting redundant functionality among MuRFs in cardiac muscle [157].

#### 2.5.2. MuRF-2

MuRF-2 anchors to the M-band via its association with p62 and NBR1 [23, 94]. Downregulation of MuRF-2 results in disrupted M-bands, in addition to perturbed intermediate filament and microtubule networks [169]. Consistent with the notion that MuRFs may have redundant functions in striated muscles, MuRF-2 deficient mice exhibit no apparent phenotype in the absence of physiological stress [157, 170]. Interestingly though, homozygous MuRF-1/MuRF-2 double knock-out mice exhibit loss of Type II fibers in soleus muscle as well as severe cardiac and modest skeletal muscle hypertrophy due to increased protein synthesis, although proteasomal degradation is unaffected [165, 171]. This suggests additional roles for MuRFs, possibly in transcriptional regulation and protein synthesis, which is consistent with their nuclear localization [23, 171].

#### 2.5.3. MuRF-3

MuRF-3 localizes to the M-band by heterodimerizing with MuRF-1 or MuRF-2 [163]. Homozygous deletion of MuRF-3 in mice reveals its role in sarcomeric organization, as evidenced by increased sarcomeric length and upregulation of select proteins, including FHL-2 [172]. In spite of the increased sarcomeric length, MuRF-3 null mice do not exhibit cardiac hypertrophy in the absence of stress [172]. However, MuRF-3 null hearts are more prone to rupture following myocardial infarction [172]. Moreover, double knock-out mice of MuRF-1 and MuRF-3 exhibit skeletal myopathy and hypertrophic cardiomyopathy, as shown by accumulation of myosin at the subsarcolemma region, myofiber fragmentation, and reduced muscle contractility [158]. Interestingly, Z-disks, but not M-bands, are disrupted in the MuRF-1/MuRF-3 double knock-out mice [158]. Since MuRF-2 is present at M-bands, but not Z-disks, it is possible that it may compensate for the loss of MuRF-1 and MuRF-3 with regards to protein turnover, thus contributing to the maintenance of M-band organization. This is consistent with MuRF proteins having redundant functions, at least partially, and highlights the importance of regulated proteasomal degradation in myofilament organization and contractility.

In addition to the MuRF family, another E3 ubiquitin ligase, cullin-3, is involved in the proteasomal degradation of small Ankyrin 1 (sAnk1), an integral membrane protein of the network sarcoplasmic reticulum that overlies M-bands and Z-disks [66]. However, since cullin-3 is localized at Z-disks rather than M-bands [66], it is highly possible that this process takes place at the former rather than the latter structure.

## 3. Cardiac and Skeletal Myopathies Associated with M-Band Proteins

**Figure 2:**
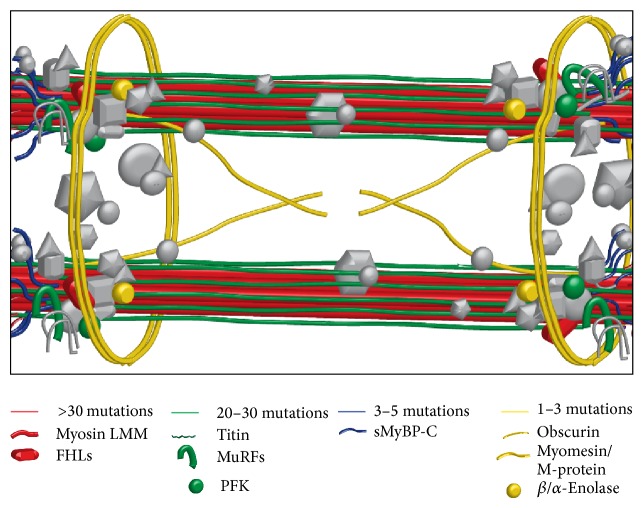
M-region proteins associated with skeletal and cardiac myopathies. Schematic representation of sarcomeric M-region proteins linked to the development of skeletal and cardiac myopathies. Proteins exhibiting no known disease-linked mutations are shown in grey color.

A significant percentage of skeletal and cardiac myopathies are linked to genetic mutations in genes encoding sarcomeric, metabolic, and enzymatic proteins [173–176], many of which are localized to the M-region (Figure 2). Herein, we present a comprehensive overview of mutations associated with sarcomeric proteins of the M-region. In particular, we summarize early and current literature on genes encoding M-region related proteins that are heavily mutated (referring the reader to focused reviews) (Tables 2 and 3) and emphasize the emerging roles of genes recently implicated in the development of skeletal and cardiac myopathies.

### 3.1. Cytoskeletal Proteins and M-Band Myopathies

#### 3.1.1. Light Meromyosin

Hereditary myosin myopathies are a group of diseases caused by mutations in the heavy chain of myosin (MyHC) [259]. Mutations have been reported in the genes encoding three muscle-specific MyHC isoforms, including* MYH7*, which is expressed in slow-twitch skeletal and cardiac muscles,* MYH3*, which is abundantly expressed in embryonic skeletal muscles, and* MYH6*, which is selectively expressed in cardiac muscle [98]. Although multiple mutations have been identified along the entire length of MyHC [174, 214, 259], we only note those associated with the light meromyosin (LMM) portion of myosin that localizes to the M-band (Table 2). Over 86 disease-causing LMM mutations have been linked to the development of different skeletal and cardiac myopathies. Of those mutations, 78 map to* MYH7* with 68 being missense mutations, 9 being insertions and/or deletions, and 1 being a nonsense mutation. Currently, the molecular alterations underlying the majority of these mutations are elusive.

#### 3.1.2. Titin

Due to the development of next-generation sequencing, routine analysis of the giant titin gene* (TTN) *has been made possible. Consequently,* TTN* has emerged as a “hot spot” for inherited skeletal and cardiac myopathies affecting mankind [175]. Over 120 disease-causing titin mutations have been reported in patients with different skeletal and cardiac myopathies [175]. Of those mutations, 23 map to the M-band region of titin, a significant percentage given the relatively small size of titin's M-band region (~200 kDa) compared to the rest of the molecule (~2-3 MDa). Of these 23 mutations, 9 are frameshift, 3 are nonsense, 6 are missense, and 5 are insertions and/or deletions (Table 2). While the mutations associated with the development of cardiomyopathies affect several domains throughout the M-band portion of titin, the ones that are linked to skeletal myopathies are mainly contained within the last Ig domain of titin, MIg10 [260].

#### 3.1.3. Obscurin

Mutations in* OBSCN*, the gene encoding giant obscurins, have been linked to the development of hypertrophic cardiomyopathy (HCM) [13, 261]. Specifically, the presence of a missense mutation results in an R4344Q substitution within the Ig58 domain of obscurin [235].* In vitro *studies have shown that the R4344Q mutation results in decreased binding of obscurin Ig58/Ig59 domains to the titin Ig9/Ig10 domains, which localize at the Z/I junction. However, the pathological effects of this mutation on sarcomeric assembly or Ca^2+^ homeostasis are still unknown.

#### 3.1.4. Myosin Binding Protein-C Slow

*MYBPC1* encodes the slow isoform of MyBP-C and has been directly implicated in the development of severe and lethal skeletal myopathies [10, 195, 196]. Two autosomal dominant missense mutations have been identified to date, W236R and Y856H, which are linked to the development of distal arthrogryposis type-1 (DA1), a severe skeletal myopathy selectively affecting distal muscles [195]. Recent work from our group has demonstrated that the W236R and Y856H mutations abolish the ability of the NH_2_ and COOH termini, respectively, to bind native actin and/or myosin and to regulate the formation of actomyosin cross-bridges* in vitro* [262]. Moreover,* MYBC1* has been causally associated with the development of lethal congenital contractual syndrome type-4 (LCCS4), a neonatal lethal form of arthrogryposis myopathy [196] that most likely results in a null phenotype. A homozygous nonsense mutation in the C2 domain of sMyBP-C (R318Stop) consists of the molecular basis of LCCS4. Given that all three mutations are encoded by exons that are constitutively expressed, they are present in all sMyBP-C variants, including those that carry a unique COOH-terminal insertion and preferentially localize to the periphery of the M-band [10, 263].

#### 3.1.5. Myomesin

The gene that encodes myomesin,* MYOM1,* has been directly linked to the development of HCM [234]. Specifically, a missense mutation resulting in V1490I substitution within the COOH-terminal Ig12 domain reduces the ability of myomesin to homodimerize (Table 2). Although the molecular etiology of HCM due to the V1490I substitution is still unknown, it is likely that mutant myomesin fails to cross-link myosin thick filaments at the M-band [234].

In addition, myomesin has been associated with myotonic dystrophy type 1 (DM1), a multisystem disease characterized by myotonia, muscle weakness, cardiac conduction defects, insulin resistance, and mental retardation. DM1 is caused by expansion of a CTG repeat present in the 3′ UTR region of dystrophia myotonica-protein kinase, DMPK [264], resulting in aggregation of muscle blind-like (MBNL) protein. MBNL is an RNA binding protein that regulates the alternative splicing of the* MYOM1 *gene [233]. Specifically, exon 17a encoding the EH-motif that links the third and fourth FnIII domains of myomesin is developmentally regulated. In normalcy, exon 17a is expressed in embryonic heart, while in heart failure it is also expressed in adult myocardium, as a compensatory mechanism to render the heart muscle more compliant [265, 266]. In the case of DM1 patients, loss of MBNL function leads to inclusion of exon 17a in adult cardiac muscle resulting in expression of a myomesin form that compromises the ability of the afflicted muscles to withstand stress and generate force [233].

#### 3.1.6. Four and a Half LIM (FHL) Domain Proteins

FHL-1 and FHL-2 have been directly linked to the development of skeletal and cardiac myopathies [176, 267]. Mutations in the* FHL1* gene, encoding FHL-1, have been linked to the development of five distinct skeletal muscle diseases, including reducing body myopathy (RBM), X-linked myopathy with postural muscle atrophy (XMPMA), scapuloperoneal myopathy (SPM), rigid spine syndrome (RSS), and Emery-Dreifuss muscular dystrophy (EDMD) [176]. Since a number of thorough reviews on FHL-1 associated myopathies have been published prior to 2011 [176, 184, 267], we will focus our discussion on new information, originating after 2011. For consistency purposes, a complete listing of all known FHL-1 mutations to date is listed in Table 2.

Since 2011, six additional mutations in* FHL-1* have been linked to the development of RBM and EDMD, increasing the total number of FHL-1 skeletal myopathy linked mutations from 29 prior to 2011 to 35 after 2011. Specifically, RBM linked mutation R95W is located within the linker region between LIM domains 1 and 2, while C104Y, H123R, C126Y, and C153S/W substitutions are present in LIM domain 2 [178, 180, 268]. Moreover, deletion of exon 6 resulting in loss of full length FHL-1 was linked to the development of EDMD [189]. Recently, Binder et al. demonstrated that patients, who presented with XMPMA due to mutations in the* FHL1 *gene, also suffer from reduced cardiac function [269]. This was observed in hemizygote males as well as heterozygote females carrying one of the following mutations, C224W, H246Y, V280M, and A168fsX195 [269].

Furthermore, six novel mutations in* FHL-1* have been directly linked with the development of HCM, by generating truncated or deleterious FHL-1 proteins. These include 2 frameshift mutations at residues 45 and 200 within LIM domains 1 and 3, respectively, 2 nonsense mutations generating premature termination codons at residues 153 and 198 within LIM domains 2 and 3, respectively, and 2 missense mutations, C209R and C276S, both located within LIM domain 3 [177, 191, 192]. In addition to the expression of truncated or poisonous forms of FHL-1, the levels of FHL-1 are also altered in cardiomyopathies. In particular, patients with HCM exhibit ~2-fold increased expression of* FHL1,* with a subsequent increase in protein levels of FHL-1 [270], while patients diagnosed with end-stage dilated cardiomyopathy (DCM) show a ~3.5-fold decrease in the levels of FHL-1 transcripts resulting in reduced protein expression [271].

Although mutations in FHL-1 protein are commonly linked to the development of skeletal and cardiac myopathies, we are just beginning to unravel the molecular mechanisms underlying the pathogenesis of these myopathies. Recently, Wilding et al. showed that select RBM (H123Y, C132F, and C153Y), SPM (W122S), and XMPMA (F127 ins 128I and C224W) FHL-1 mutants accumulate in reducing bodies or protein aggregates when overexpressed in C2C12 cells [272]. These reducing bodies are phenotypically similar to those found in patients suffering from the corresponding diseases. In addition, these same mutations result in impaired myoblast differentiation when overexpressed in C2C12 cells, consistent with the loss of normal FHL-1 function [272]. Conversely, select EDMD (K157VfsX36, C273LfsX11, C276Y, and E281Stop) and EDMD/HCM (C209R) FHL-1 mutations result in reduced protein expression when overexpressed in C2C12 cells, suggesting impaired transcriptional regulation and/or protein stability and degradation [272]. Thus, these studies are the first to suggest potential molecular alterations that underlie the different FHL-1 linked myopathies.

FHL-2 has also been associated with heart failure progression and the development of DCM. A missense mutation, G48S, in the first LIM domain of FHL-2 has been identified in a patient with familial DCM [194] (Table 2). The presence of this mutation reduces the ability of mutant FHL-2 protein to bind titin, suggesting a structural role for FHL-2 at the M-band.

### 3.2. Metabolic Enzymes and M-Band Myopathies

In addition to alterations in genes encoding cytoskeletal proteins, mutations in the metabolic enzymes PFK, *β*-enolase, and AMPD that localize at the M-band have been linked to the development of skeletal and cardiac myopathies; this group of diseases is collectively classified as “metabolic myopathies.” Affected individuals present with muscle weakness, occasionally triggered by exercise, chronic respiratory failure, muscle rigidity, and decreased voluntary contractions [273]. Tarui disease, also referred to as glycogen storage disease (GSD) type VII, is a rare disorder involving impaired glycogen metabolism due to PFK deficiency and is characterized by exercise intolerance, myalgias, muscle cramps, and episodic myoglobinuria [252]. To date, 21 mutations in the* PFK *gene have been linked to the development of Tarui/GSD type VII disease (Table 3) [252, 255] (for a thorough discussion on Tarui disease and the molecular details of the identified mutations, please see [252]). Another form of GSD, referred to as GSD type XIII, is linked to defects in *β*-enolase. Two missense mutations (G156N and G374E) in the *β*-enolase gene have been linked to the development of GSD type XIII. These mutations result in decreased protein levels leading to a dramatic reduction (~95%) in cellular enolase activity [248] (Table 3).

Lastly, missense and truncation mutations in the human* AMPD1* gene have been linked with AMPD deficiency in skeletal muscle [274–277]. Specifically, the Q12X nonsense mutation gives rise to a premature stop codon leading to the generation of truncated mRNA and loss of AMPD-1 protein [275]. In addition, the P48L, Q156H, K287I, R388W, and R425H missense mutations result in expression of mutant AMPD-1 proteins with negligible enzymatic activity [274, 276, 277], while a deletion mutation (IVS2 del CTTT) leads to expression of multiple inactive spliced forms of the protein [277]. While loss of AMPD-1 may be partially compensated by isoforms encoded by* AMPD3* [278], the importance of AMPD-1 in maintaining optimal AMP levels is underscored by the severe effects of many of these disease-linked mutations.

### 3.3. Ubiquitin Ligases and M-Band Myopathies

#### 3.3.1. MuRFs

It is only very recently that mutations in members of the MuRF family have been linked to the development of hypertrophic cardiomyopathies. Su et al. recently identified 8, 12, and 10 mutations in MuRF-1, MuRF-2, and MuRF-3 genes, respectively (Table 3) [249]. Interestingly, these mutations are suggested to modify the severity of the HCM phenotype but not cause it* per se*. Moreover, select HCM linked MuRF-1 and MuRF-2 mutations cosegregate with mutations in genes encoding sarcomeric proteins, that is,* MYH7, MYBPC3, MYC2*, and* MYL3*. However, the contribution of these MuRF-1 and MuRF-2 mutations in the pathogenesis of HCM is still unknown.

In addition, the expression profile of MuRF-1 is differentially regulated in response to pathophysiological processes, such as aging, atrophy, and senescence. Specifically, the levels of MuRF-1 are significantly increased in the initial phases of muscle disuse and atrophy in humans; this is consistent with a decrease in muscle fiber size [279]. Conversely, in aged skeletal muscle, the levels of MuRF-1 are decreased, coinciding with slowing of muscle atrophy [279]. Moreover, muscle loss is often associated with chronic diseases, such as cirrhosis and heart failure. Muscle biopsies obtained from malnourished cirrhotic patients exhibiting muscle atrophy contain increased amounts of MuRF-1 [280]. Similarly, MuRF-1's expression is upregulated in skeletal muscles of patients with chronic heart failure [281]. However, this trend is reversed in the same patients following exercise training [281].

Although the molecular etiologies underlying the differential expression of the MuRF proteins in skeletal and cardiac myopathies are yet to be defined, it is apparent that they play key roles in regulating muscle fiber size and muscle loss [282].

## 4. Perspectives and Future Directions

Throughout the last decade, it has become clear that in addition to its structural role, the M-region also acts as a mechanosensor, signal transduction center, and metabolic hub (Figure 1 and Table 1). Distinct mutations or alterations in the expression levels of M-band proteins have been implicated in the development of skeletal and cardiac muscle diseases. To date, more than 210 distinct mutations affect proteins that localize to the M-region. Notably, a striking 163 missense and nonsense mutations, 21 frameshift mutations, and 26 deletions/insertions within genes encoding M-region related proteins (Figure 2 and Tables 2 and 3) have been associated with the development of different forms of skeletal and cardiac myopathies. The severity of these diseases can vary dramatically, depending on the nature of the mutation and the role of the affected protein. Our current understanding of the molecular pathophysiology of individual mutations is still incomplete and only just emerging in most cases. Deciphering how these mutations alter M-band structure, contractile activity, signaling networks, posttranslational modifications, and energy production will aid in improving clinical diagnosis and developing individualized therapeutic approaches for affected individuals.

## Figures and Tables

**Table 1:** Properties of M-region proteins.

Protein		Localization	Muscle specificity	Residency	PDB ID
*M-band organization *					
Complex 1	Obscurins (ABD)	Periphery	Cardiac/skeletal	Permanent	NA
	Ankyrin-B (Exon 43′)	Periphery	Cardiac	Permanent	NA
	PP2A (B56*α*)	Periphery	Cardiac/skeletal	Permanent	NA

Complex 2	Titin (Mis4)	Periphery/interior	Cardiac/skeletal	Permanent	NA
	Bin-1	Interior	Cardiac/skeletal (developmental)	Transient	1MV3
	Cdc2 Kinase	Interior	Cardiac/skeletal (developmental)	Transient	NA

	Myomesin	Periphery/interior	Cardiac/skeletal	Permanent	3RBS, 2Y23, 2R15, 2Y25
	SmyD1	Periphery	Cardiac/fast skeletal	Permanent	3N71
	FHLs	Periphery	Cardiac/skeletal	Permanent	2EGQ, 2D8Z

*Cytoskeletal remodeling *					
Obscurins (RhoGEF)		Periphery	Cardiac/skeletal	Permanent	NA
RhoA		Periphery	Cardiac/skeletal	Transient	1LB1, 3KZ1
CRIK		Periphery	Cardiac/skeletal	Transient	NA
Active ROCK1		Periphery	Cardiac/skeletal	Transient	2ETR

*Mechanosensing *					
Titin (Titin Kinase)		Periphery/interior	Cardiac/skeletal	Permanent	4JNW, 1TKI
NBR1		Periphery	Cardiac/skeletal	Permanent	4OLE
P62		Periphery	Cardiac/skeletal	Permanent	2KTR, 3B0F, 2MGW
MuRFs		Periphery	Cardiac/skeletal	Permanent	4M3L, 3Q1D

*Metabolism *					
PFK		Periphery	Cardiac/skeletal	Permanent	4OMT
M-CK		Periphery	Cardiac/skeletal	Permanent	1I0E
AK		Periphery	Cardiac/skeletal	Permanent	2C95
Enolases		Periphery	Cardiac/skeletal	Permanent	3B97, 2XSX
AMPD		Periphery	Cardiac/skeletal	Permanent	2Y2C

*Proteasomal degradation *					
MuRFs		Periphery	Cardiac/skeletal	Permanent	4M3L, 3Q1D

Note: protein domains mediating complex formation or participating in cellular processes are shown in parenthesis when known. Acronyms of proteins are described in the text; ABD: ankyrin binding domain; NA: not available. The PDB files of proteins in Complex 1 and Complex 2, as well as obscurins (RhoGEF) and titin (titin kinase), are associated with the specific domains that mediate binding within the complex; in all other cases, the available PDB files for the entire protein are provided.

**Table 2:** Disease-causing mutations in genes encoding structural proteins of the M-region.

Protein	Mutation	Region on protein	Effect	Disease	Reference
FHL-1	K45SfsX1	LIM domain 1	Unknown	HCM	[177]
FHL-1	R95W	Linker region between LIM domain 1 and 2	Unknown	RBM	[178]
FHL-1	C101F	LIM domain 2	Unknown	RBM	[179]
FHL-1	102–104 del KFC	LIM domain 2	Unknown	RBM	[179]
FHL-1	C104R/Y	LIM domain 2	Unknown	RBM	[179, 180]
FHL-1	111–229 del ins G	LIM domain 2	Unknown	EDMD	[181]
FHL-1	N112FfsX51	LIM domain 2	Unknown	EDMD	[181]
FHL-1	W122S/C	LIM domain 2	Unknown	SPM	[182, 183]
FHL-1	H123Y/Q/L/R	LIM domain 2	Unknown	RBM	[178, 184–186]
FHL-1	K124RfsX6	LIM domain 2	Unknown	EDMD	[181]
FHL-1	F127 ins 128I	LIM domain 2	Unknown	XMPMA	[187]
FHL-1	C132F	LIM domain 2	Unknown	RBM	[186]
FHL-1	C150Y/R/S	LIM domain 2	Unknown	RBM	[179, 185, 188]
FHL-1	151–153 del VTC	LIM domain 2	Unknown	RSS	[179]
FHL-1	C153Y/R/S/W	LIM domain 2	Unknown	RBM	[186]
FHL-1	153Stop	LIM domain 2	Unknown	HCM	[177]
FHL-1	Delete exon 6 ins 84 bp	LIM domain 3	Loss of full length FHL-1A, increase in FHL-1C	EDMD	[189]
FHL-1	K157VfsX36	LIM domain 3	Unknown	EDMD	[181]
FHL-1	A168GfsX195	LIM domain 3	Unknown	XMPMA	[187]
FHL-1	194Stop	LIM domain 3	Premature stop codon and truncated protein corresponding to FHL-1C	XMPMA	[190]
FHL-1	198Stop	LIM domain 3	Unknown	HCM	[191]
FHL-1	F200fs32X	LIM domain 3	Unknown	HCM	[192]
FHL-1	C209R	LIM domain 3	Unknown	EDMD/HCM	[181, 193]
FHL-1	C224W	LIM domain 4	Unknown	XMPMA	[187]
FHL-1	H246Y	LIM domain 4	Unknown	XMPMA	[190]
FHL-1	C273LfsX11	LIM domain 4	Unknown	EDMD	[181]
FHL-1	C276Y	LIM domain 4	Unknown	EDMD	[181]
FHL-1	C276S	LIM domain 4	Unknown	HCM	[177]
FHL-1	V280M	NLS of FHL-1B	Unknown	XMPMA	[190]
FHL-1	E281Stop	Extreme COOH-terminus	Unknown	EDMD	[181]
FHL-2	G48S	LIM domain 1	Loss of titin binding	DCM	[194]
sMyBP-C	W236R	M-motif	Loss of actin and myosin binding	DA-1	[195]
sMyBP-C	R318Stop	IgC2	Premature stop codon and truncated protein	LCCS4	[196]
sMyBP-C	Y856H	IgC8	Loss of myosin binding	DA-1	[195]
MyH 3	841-841 del L	LMM	Reduced catalytic activity	DA Sheldon-Hall syndrome	[197]
MyH 6	A1004S E1457K	LMM	Unknown	DCM	[198]
MyH 6	Q1065H	LMM	Unknown	HCM	[198]
MyH 6	R1116S A1366D A1443D R1865Q	LMM	Unknown	CHD	[199]
MyH 7	847-847 del K	LMM	Unknown	HCM	[200]
MyH 7	M852T	LMM	Unknown	HCM	[201]
MyH 7	R858C	LMM	Unknown	HCM	[200]
MyH 7	R869G	LMM	Unknown	HCM	[201]
MyH 7	R870H	LMM	Unknown	HCM	[202]
MyH 7	883-883 del E	LMM	Unknown	HCM	[201]
MyH 7	E894G	LMM	Unknown	HCM	[200]
MyH 7	D906G	LMM	Unknown	HCM	[203]
MyH 7	L908V	LMM	Unknown	HCM with CCD	[204]
MyH 7	E921K	LMM	Unknown	HCM	[200]
MyH 7	E924K E949K	LMM	Unknown	HCM	[205]
MyH 7	D928V	LMM	Unknown	HCM	[206]
MyH 7	E931K	LMM	Unknown	HCM	[200]
MyH 7	E935K	LMM	Unknown	HCM	[207]
MyH 7	D953H	LMM	Unknown	HCM	[200]
MyH 7	T1019N	LMM	Unknown	DCM	[208]
MyH 7	R1053Q	LMM	Unknown	HCM	[209]
MyH 7	G1057S	LMM	Unknown	HCM	[200]
MyH 7	L1135R	LMM	Unknown	HCM	[201]
MyH 7	R1193S	LMM	Unknown	DCM	[208]
MyH 7	E1218Q	LMM	Unknown	HCM	[201]
MyH 7	N1327K	LMM	Reduced *α*-helical content of the rod domain	HCM	[210]
MyH 7	E1356K	LMM	Reduced *α*-helical content of the rod domain	HCM	[211]
MyH 7	E1377M A1379T R1382W	LMM	Unknown	HCM	[201]
MyH 7	R1420W	LMM	Unknown	HCM	[200]
MyH 7	E1426K	LMM	Unknown	DCM	[208]
MyH 7	A1439P	LMM	Unknown	MPD1	[212]
MyH 7	K1459N	LMM	Unknown	HCM	[200]
MyH 7	L1467V	LMM	Unknown	Congenital myopathy	[213]
MyH 7	L1481P	LMM	Unknown	MPD1	[214]
MyH 7	R1500W	LMM	Reduced *α*-helical content of the rod domain	DCM	[215]
MyH 7	R1500P 1617-1617 del K	LMM	Unknown	Laing distal myopathy	[216]
MyH 7	1508-1508 del E	LMM	Unknown	MPD1	[217]
MyH 7	T1513S	LMM	Unknown	HCM	[200]
MyH 7	Q1541P	LMM	Unknown	MPD1	[214]
MyH 7	E1555K	LMM	Reduced *α*-helical content of the rod domain	HCM	[218]
MyH 7	R1588P	LMM	Unknown	MPD1	[213]
MyH 7	L1591P	LMM	Unknown	MPD1	[219]
MyH 7	L1597R	LMM	Unknown	Axial myopathy, contractual myopathy	[220]
MyH 7	T1599P	LMM	Unknown	MPD1	[214]
MyH 7	A1603P	LMM	Unknown	MPD1	[217]
MyH 7	R1608P	LMM	Unknown	Congenital myopathy, HCM	[214]
MyH 7	L1612P	LMM	Unknown	MPD1	[214]
MyH 7	1617-1617 del K	LMM	Unknown	MPD1, DCM	[214, 216]
MyH 7	R1634S	LMM	Unknown	DCM	[208]
MyH 7	A1636P L1646P R1662P	LMM	Unknown	MPD1	[214]
MyH 7	A1663P	LMM	Unknown	MPD1	[216]
MyH 7	1669-1669 del E	LMM	Unknown	MPD1	[214]
MyH 7	V1691M	LMM	Unknown	HCM	[201]
MyH 7	L1706P	LMM	Unknown	MPD1	[216]
MyH 7	R1712W	LMM	Unknown	HCM	[210]
MyH 7	L1723P	LMM	Unknown	CCD	[221]
MyH 7	1729-1729 del K	LMM	Unknown	Laing distal myopathy	[216]
MyH 7	E1753K	LMM	Unknown	HCM	[210]
MyH 7	A1766T	LMM	Unknown	LVNC	[222]
MyH 7	E1768K	LMM	Increased *α*-helical content of the rod domain	HCM	[200]
MyH 7	S1776G	LMM	Unknown	HCM	[223]
MyH 7	A1777T	LMM	Unknown	HCM	[201]
MyH 7	1784-1784 del K	LMM	Unknown	MPD1, MSM	[219, 224]
MyH 7	L1793P	LMM	Destabilization of the thick filaments	HCM with MSD	[225, 226]
MyH 7	1793-1793 del L	LMM	Unknown	MPD1	[214]
MyH 7	E1801K	LMM	Unknown	MPD1, DCM, HCM	[214, 217]
MyH 7	T1834M	LMM	Unknown	HCM	[200]
MyH 7	R1845W	LMM	Alters interactions between filaments	MSM	[227]
MyH 7	E1856K	LMM	Unknown	Late onset myopathy with cardiac involvement	[228]
MyH 7	E1883K	LMM	Destabilization of the thick filaments	HCM	[226, 229]
MyH 7	H1901L	LMM	Alters interactions between filaments	MSM	[230]
MyH 7	E1914K	LMM	Unknown	DCM	[214]
MyH 7	N1918K	LMM	Unknown	LVNC	[231]
MyH 7	T1929M	LMM	Unknown	HCM	[200]
MyH 7	Stop1936W	LMM	Unknown	MSM	[232]
Myomesin	Aberrant splicing of exon 17a	EH-motif	Premature stop codon and truncated protein	MD1	[233]
Myomesin	V1490I	Ig12	Reduced dimerization	HCM	[234]
Obscurin	R4344Q	Ig58	Loss of titin binding	HCM	[235]
Titin	S33705LfsX4	TK	Unknown	LGMD2J	[236]
Titin	N34020TfsX9	TK	Increased structural stability of TK, loss of interactions with proteins partners of TK	MmD-HD	[175]
Titin	R34091W	TK	Unknown	HMERF	[23]
Titin	R34175Stop	MIg1	Unknown	MmD-HD	[175]
Titin	32664-32665 del ins K	MIg2	Unknown	HCM	[237]
Titin	P34617QinsX3	MIs2	Unknown	CNM	[238]
Titin	R34637Q	MIg4	Unknown	DCM	[239]
Titin	A32606fsX7	MIg5	Unknown	DCM	[240]
Titin	Q35176HfsX9	MIg5	Truncated titin	MmD-HD (EOMFC)	[241]
Titin	Q35278Stop	MIs4	Unknown	MmD-HD	[175]
Titin	G35340VfsX65	MIg6	Unknown	CNM	[238]
Titin	33710-33711 del ins K	MIg6	Unknown	HCM	[237]
Titin	S35469SfsX11	MIg7	Unknown	MmD-HD	[175]
Titin	K35524RfsX22	MIs6	Unknown	MmD-HD (EOMFC)	[241]
Titin	32986-32987 del ins K	MIg8	Unknown	DCM	[240]
Titin	M35859T	MIs7	Unknown	ARVC	[242]
Titin	S35883QfsX10	MIs7	Unknown	TMD	[243]
Titin	Q35927–35931W del ins VKQK	MIg10	Truncated titin	TMD, LGMD2J, MD	[236, 244, 245]
Titin	H35946P	MIg10	Unknown	TMD	[246]
Titin	I35947N	MIg10	Unknown	TMD	[247]
Titin	L35956P	MIg10	Unknown	TMD	[244]
Titin	K35963NfsX9	MIg10	Unknown	TMD, CNM	[238, 243]
Titin	Q35964Stop	MIg10	Truncated titin	TMD	[243]

Note: nomenclature refers to the canonical full-length human isoforms; FHL-1, NP_001153174.1, sMyBP-C, AAI43503.1, MyH 3, NP_002461.2, MyH 6, NP_002462.2, MyH 7, NP_000248.2, myomesin, CAF18565.1, obscurin, CAC44768.1, titin, NP_001254479.2. HCM: hypertrophic cardiomyopathy, RBM: reducing body myopathy, XMPMA: X-linked myopathy with postural muscle atrophy, SPM: scapuloperoneal myopathy, RSS: rigid spine syndrome, EDMD: Emery-Dreifuss muscular dystrophy, DCM: dilated cardiomyopathy, DA-1: distal arthrogryposis type 1, LCCS4: lethal congenital contracture syndrome type 4, MPD1: Laing distal myopathy, CHD: congenital heart defect, CCD: central core disease, MSM: myosin storage myopathy, LVNC: left ventricular noncompaction, MD1: myotonic dystrophy type 1, LGMD2J: limb-girdle muscular dystrophy type 2J, MmD-HD: multiminicore disease with heart disease, HMERF: hereditary myopathy with early respiratory failure, CNM: centronuclear myopathy, EOMFC: early-onset myopathy with fatal cardiomyopathy, ARVC: arrhythmogenic right ventricular cardiomyopathy, TMD: tibial muscular dystrophy, MD: muscle disease, NLS: nuclear localization sequence, TK: titin kinase, MIgX: titin M-band IgX, MyH: myosin heavy chain, and LMM: light meromyosin.

**Table 3:** Proteins with enzymatic activity at the M-region and related diseases.

Protein	Mutation	Effect	Disease	Reference
*β*-Enolase	G156N G374E	Unknown	GSD XIII	[248]
MuRF1	S5L	Unknown	HCM	[249]
MuRF1	F73S	Unknown	HCM	[249]
MuRF1	R86C/H	Unknown	HCM	[249]
MuRF1	I101F	Unknown	HCM	[249]
MuRF1	T232M	Unknown	HCM	[249]
MuRF1	E299Stop	Unknown	HCM	[249]
MuRF1	M305I	Unknown	HCM	[249]
MuRF1	A318D	Unknown	HCM	[249]
MuRF2	C50Y	Unknown	HCM	[249]
MuRF2	P79A	Unknown	HCM	[249]
MuRF2	Q187fs	Unknown	HCM	[249]
MuRF2	L241M	Unknown	HCM	[249]
MuRF2	S252F	Unknown	HCM	[249]
MuRF2	E371fs	Unknown	HCM	[249]
MuRF2	P392T	Unknown	HCM	[249]
MuRF2	K425N	Unknown	HCM	[249]
MuRF2	A488T	Unknown	HCM	[249]
MuRF2	T506S	Unknown	HCM	[249]
MuRF2	H523W	Unknown	HCM	[249]
MuRF2	F538fs	Unknown	HCM	[249]
MuRF3	P9L	Unknown	HCM	[249]
MuRF3	G94C	Unknown	HCM	[249]
MuRF3	P115S	Unknown	HCM	[249]
MuRF3	L163P	Unknown	HCM	[249]
MuRF3	A221V	Unknown	HCM	[249]
MuRF3	R249Q	Unknown	HCM	[249]
MuRF3	R269H	Unknown	HCM	[249]
MuRF3	K270N	Unknown	HCM	[249]
MuRF3	P346T	Unknown	HCM	[249]
MuRF3	G373D	Unknown	HCM	[249]
PFK	R39P/L	Unknown	GSD VII	[250, 251]
PFK	G57V	Unknown	GSD VII	[252]
PFK	G80fs4X	Unknown	GSD VII	[252]
PFK	R95Stop	Unknown	GSD VII	[253]
PFK	R100Q	Unknown	GSD VII	[254]
PFK	S108C	Unknown	GSD VII	[252]
PFK	G209D	Unknown	GSD VII	[254]
PFK	N309G	Unknown	GSD VII	[255]
PFK	D543A	Unknown	GSD VII	[250]
PFK	D591A	Unknown	GSD VII	[252]
PFK	P668Q	Unknown	GSD VII	[251]
PFK	W686C	Unknown	GSD VII	[254]
PFK	R696H	Unknown	GSD VII	[251]
PFK	78 bp del exon 5	Unknown	GSD VII	[254]
PFK	5 or 12 bp del exon 7	Unknown	GSD VII	[250]
PFK	75 bp del exon 15	Unknown	GSD VII	[256]
PFK	Retention of intron 13, truncated protein	Unknown	GSD VII	[257]
PFK	Retention of intron 16, truncated protein	Unknown	GSD VII	[257]
PFK	169 bp del exon 19	Unknown	GSD VII	[258]

Note: nomenclature refers to the canonical full-length human isoforms; beta-enolase, NP_001967.3, MuRF1, NP_115977.2, MuRF2, Q9BYV6.2, MuRF3, NP_912730.2, PFK, and NP_000280.1. HCM: hypertrophic cardiomyopathy; GSD: glycogen storage disease.
